# Congenital Rubella Syndrome in Child of Woman without Known Risk Factors, New Jersey, USA

**DOI:** 10.3201/eid2002.131233

**Published:** 2014-02

**Authors:** Samantha I. Pitts, Gregory S. Wallace, Barbara Montana, Elizabeth F. Handschur, Debrah Meislich, Alethia C. Sampson, Suzanne Canuso, Jennifer Horner, Albert E. Barskey, Emily S. Abernathy, Joseph P. Icenogle

**Affiliations:** Johns Hopkins University School of Medicine, Baltimore, Maryland, USA (S.I. Pitts);; New Jersey Department of Health, Trenton, New Jersey, USA (S.I. Pitts, B. Montana, E.F. Handschur, S. Canuso);; Council of State and Territorial Epidemiologists, Atlanta, Georgia, USA (S.I. Pitts);; Centers for Disease Control and Prevention, Atlanta (G.S. Wallace, A.E. Barskey, E.S. Abernathy, J.P. Icenogle);; Cooper Medical School of Rowan University, Camden, New Jersey, USA (D. Meislich);; Burlington County Health Department, Westampton, New Jersey, USA (A.C. Sampson, J. Horner)

**Keywords:** Rubella syndrome, congenital rubella syndrome, rubella, viruses, congenital, vaccination, contact tracing, case reports, New Jersey, USA

## Abstract

We report a case of congenital rubella syndrome in a child born to a vaccinated New Jersey woman who had not traveled internationally. Although rubella and congenital rubella syndrome have been eliminated from the United States, clinicians should remain vigilant and immediately notify public health authorities when either is suspected.

Once a main cause of congenital abnormalities (*1*), congenital rubella syndrome (CRS) is now rare in the United States. However, rubella remains a common illness in countries without robust vaccination programs. Since rubella was declared eliminated in the United States in 2004 (*2*), 6 cases of CRS have been reported to the Centers for Disease Control and Prevention (CDC); 5 were likely imported cases (*3*). We describe the sixth case.

## Case Report

In 2008, a full-term boy was born with microcephaly and a petechial rash. The infant’s US-born mother reported no rash illness, travel history, or known contact with ill persons during the first 4 months of pregnancy. She lived with her 2 other children and the case-patient’s father, who was born in Brazil and had not traveled internationally during the mother’s pregnancy.

In 2002, the case-patient’s mother had an equivocal rubella virus IgG titer of 5 (nonimmune <5) and in 2003 received measles, mumps, and rubella (MMR) vaccine. Vaccination status of the father was unknown. Maternal rubella virus IgG titer at 4 months’ gestation was >400 IU/mL (immune >9).

The infant was delivered by urgent cesarean section because of cardiac decelerations during labor. Apgar scores at 1 and 5 minutes were 9 (of 10 total). At birth, the child weighed 2.7 kg (10th percentile), and he was 48.25 cm (15th percentile) in length and had a head circumference of 31.75 cm (<3rd percentile). He had a petechial rash on the face, back, and upper extremities and a systolic heart murmur. No jaundice or hepatosplenomegaly was noted.

Initial tests showed a leukocyte count of 13.8 × 10^9^/L (reference 9–30) with atypical lymphocytes; a hemoglobin level of 202 g/L (reference 135−195); abnormal erythrocyte morphology, including macrocytosis, polychromasia, and poikilocytosis; and a platelet count of 98,000 × 10^9^/L (reference 140–440). Computed tomographic scan of the head revealed 2 small calcifications in the left corona radiata. Results of neonatal hearing screening were normal. Urine samples were cultured for cytomegalovirus (CMV), and serum samples were tested for toxoplasma and parvovirus IgM; all results were negative. The infant was discharged from the hospital on day 4 with a suspected congenital infection.

At 16 days of age, the child was seen by an infectious disease physician. Examination revealed hepatosplenomegaly, and the systolic heart murmur was detected across the precordium, with radiation to the back. CMV quantitative PCR and testing for lymphocytic choriomeningitis IgM and IgG were requested, but not obtained. Congenital rubella was considered, but serologic tests were not ordered. A subsequent echocardiogram demonstrated supravalvular and peripheral pulmonic stenosis, a small patent ductus arteriosis, and a patent foramen ovale.

The child was delayed in attaining developmental milestones. At 6.5 months of age, he was referred to a geneticist, who requested multiple tests, including tests for rubella, CMV, and lymphocytic choriomeningitis. Rubella virus serum IgM was 7.2 (positive >1.0) and IgG was 59 IU/mL (immune >9). The local health department received notification of the test results and immediately initiated an investigation in collaboration with the New Jersey Department of Health. During multiple interviews, the child’s family denied any exposure to international travelers or persons with a rash illness during the potential exposure period. No source contact or secondary transmission was identified. New Jersey Department of Health and the infectious disease physician requested that the child not attend day care and avoid exposure to unvaccinated infants until after his first birthday, when he would be presumed to be free of infection. MMR vaccination at 12 months of age was withheld until completion of additional diagnostic testing.

When the infant was 7.5 months of age, rubella virus RNA was detected from a nasal wash sample by using reverse transcription PCR (RT-PCR); results of a nasal wash culture and serum RT-PCR were negative. CDC culture and RT-PCR results for nasal samples collected when the infant was 10 and 12 months of age were also negative. However, ELISAs performed by CDC on a serum sample collected when the infant was 12 months of age confirmed the presence of rubella virus IgM antibodies (Diamedix Rubella IgM Capture EIA; IVAX Corp., Miami, FL, USA) and IgG antibodies (Wampole Rubella IgG ELISA II; Alere, Waltham, MA, USA); results were 2.43 and 5.9, respectively (positive >1.1).

## Conclusions

The clinical features and laboratory results for this case are most consistent with CRS, although initial testing was delayed until the infant was 6.5 months of age. Clinical findings for the case-patient (microcephaly, developmental delay, congenital heart disease) were compatible with CRS and met 3 of the Council of State and Territorial Epidemiologists’ laboratory criteria for a confirmed case of CRS (*4*) (Table).

**Table:** Criteria of the Council of State and Territorial Epidemiologists (2009) for a confirmed case of congenital rubella^*^

Criteria	Criteria met by case-patient
Clinical criteria	
Congenital heart disease†	Yes
Microcephaly	Yes
Developmental delay	Yes
Hepatosplenomegaly	Yes
Purpura	No
Cataracts/congenital glaucoma	No
Hearing impairment	No
Pigmentary retinopathy	No
Jaundice	No
Meningoencephalitis	No
Radiolucent bone disease	No
Laboratory criteria	
Demonstration of rubella IgM	Yes
Persistent rubella antibody in an infant	Yes
Positive rubella RT-PCR	Yes
Isolation of rubella virus	No

*RT-PCR, reverse transcription PCR. Source (*4*). †Usually patent ductus arteriosus or peripheral pulmonary artery stenosis.

CRS occurs rarely in children of women with a history of vaccination or documented immunity by rubella virus titer. When CRS does occur, it is frequently a result of asymptomatic rubella reinfection in the mother (*5**,**6*). In this case-patient, CRS was likely the result of 2 rare events: his mother’s lack of immunity after vaccination and her exposure to rubella in a highly immune population.

Delays in diagnostic testing and reporting hindered the public health contact investigation of this case. Because the investigation began ≈1 year after the maternal exposure period, the persons involved had limited recall. Earlier diagnosis and reporting could have improved our contact investigation and provided for earlier isolation of the case-patient.

We were unable to identify a maternal rubella exposure. Given rubella’s elimination from the United States, we speculate that the case-patient’s mother may have had contact with an international traveler from a region with circulating rubella. In the years following documentation of rubella elimination in the United States, surveillance has identified a small number of circumstances in which foreign visitors without confirmed rubella transmitted the virus to US residents.

Several features of the laboratory testing bear further examination. The persistence of rubella IgM at 12 months has previously been reported in CRS cases (*7*). The child’s rubella IgG level did not decline as would be expected as a result of the loss of maternal antibody; this lack of decline is consistent with rubella infection. In addition, the case-patient’s serologic results were most consistent with CRS, given his clinical features, his low risk for postnatal rubella infection, and the fact that he was not vaccinated. When the infant was 7.5 months of age, RT-PCR was positive for 1 of 2 specimens. However, when the infant was 10 and 12 months of age, RT-PCR was negative, as were culture results when the infant was 7.5 and 10 months of age. These findings are consistent with the diagnosis of CRS because for ≈50% of children with CRS, culture results are negative by 3 months of age (*8*).

We found no evidence of secondary rubella transmission. A model has suggested that to interrupt rubella transmission, >87.5% of the population must be immune (*9*). On the basis of the 2010 National Immunization Survey, 86.1% (95% CI 80.4%–91.8%) of New Jersey children 19–35 months of age had received >1 doses of MMR vaccine, and 92.6% (95% CI 89.1%–96.1%) of adolescents 13–17 years of age had received >2 doses (*10*,*11*). Continued rubella elimination relies upon the maintenance of high levels of immunity in the population; thus, public health professionals should continue to strive to achieve high levels of rubella immunization in their communities.

Despite its elimination from the United States, rubella infection and CRS continue to occur rarely: 6 cases of CRS have been reported to CDC since 2004. Clinicians should remain vigilant to the possibility of rubella and CRS and immediately notify public health authorities when either is suspected.
